# Expression and functional implications of YME1L in nasopharyngeal carcinoma

**DOI:** 10.1038/s41419-024-06811-6

**Published:** 2024-06-18

**Authors:** Fuwei Cheng, Haiping Huang, Shiyao Yin, Ji-Sheng Liu, Peng Sun

**Affiliations:** Department of Otolaryngology, The First Affiliated Hospital of Soochow University, Suzhou, China

**Keywords:** Oral cancer, Targeted therapies

## Abstract

Mitochondria play a crucial role in the progression of nasopharyngeal carcinoma (NPC). YME1L, a member of the AAA ATPase family, is a key regulator of mitochondrial function and has been implicated in various cellular processes and diseases. This study investigates the expression and functional significance of YME1L in NPC. YME1L exhibits significant upregulation in NPC tissues from patients and across various primary human NPC cells, while its expression remains relatively low in adjacent normal tissues and primary nasal epithelial cells. Employing genetic silencing through the shRNA strategy or knockout (KO) via the CRISPR-sgRNA method, we demonstrated that YME1L depletion disrupted mitochondrial function, leading to mitochondrial depolarization, reactive oxygen species (ROS) generation, lipid peroxidation, and ATP reduction within primary NPC cells. Additionally, YME1L silencing or KO substantially impeded cell viability, proliferation, cell cycle progression, and migratory capabilities, concomitant with an augmentation of Caspase-apoptosis activation in primary NPC cells. Conversely, ectopic YME1L expression conferred pro-tumorigenic attributes, enhancing ATP production and bolstering NPC cell proliferation and migration. Moreover, our findings illuminate the pivotal role of YME1L in Akt-mTOR activation within NPC cells, with Akt-S6K phosphorylation exhibiting a significant decline upon YME1L depletion but enhancement upon YME1L overexpression. In YME1L-silenced primary NPC cells, the introduction of a constitutively-active Akt1 mutant (caAkt1, at S473D) restored Akt-S6K phosphorylation, effectively ameliorating the inhibitory effects imposed by YME1L shRNA. In vivo studies revealed that intratumoral administration of YME1L-shRNA-expressing adeno-associated virus (AAV) curtailed subcutaneous NPC xenograft growth in nude mice. Furthermore, YME1L downregulation, concurrent with mitochondrial dysfunction and ATP reduction, oxidative injury, Akt-mTOR inactivation, and apoptosis induction were evident within YME1L-silenced NPC xenograft tissues. Collectively, these findings shed light on the notable pro-tumorigenic role by overexpressed YME1L in NPC, with a plausible mechanism involving the promotion of Akt-mTOR activation.

## Introduction

Nasopharyngeal carcinoma (NPC) is a distinct type of head and neck cancer that arises from the epithelial lining of the nasopharynx [1, 2], leading to over 70,000 death each year globally [2–4]. It is characterized by its potential for early metastasis, often diagnosed at advanced stages, making treatment extremely challenging [5–7]. Current therapeutic strategies for NPC typically encompass a multimodal approach, combining radiation therapy as the primary treatment modality with concurrent or adjuvant chemotherapy [5–7]. While these therapies have shown effectiveness in many cases, recurrent and metastatic NPC remains a clinical challenge, necessitating ongoing studies to explore novel treatment modalities and targeted therapies [5]. Targeted therapies for NPC have shown promise by focusing on specific molecular pathways and genetic aberrations driving NPC progression, including EGFR inhibitors (cetuximab) and immune checkpoint inhibitors (pembrolizumab and nivolumab) [1, 5, 8].

Mitochondria play a crucial role in the progression of NPC [9–11]. Studies have suggested that mitochondrial proteins are dysregulated in NPC and are important for cancer initiation, progression and therapy-resistance, highlighting its significance for developing therapeutic strategies targeting these proteins [9, 10, 12]. Dysfunctional mitochondria have been linked to the altered bioenergetics and metabolic reprogramming observed in NPC cells [9, 10, 12]. These aberrant mitochondrial function contribute to the enhanced survival, proliferation, invasiveness and therapy-resistance of NPC cells [9, 11–14]. Additionally, mitochondria participate in apoptotic signaling pathways, and their dysfunction can inhibit programmed cell death, thereby facilitating the survival and growth of malignant NPC cells. Understanding the interplay between mitochondria and NPC progression holds promise for developing targeted therapeutic strategies [11, 13, 14].

YME1L (YME1 Like 1 ATPase) is a pivotal member of the AAA family of ATPases, residing primarily within the inner mitochondrial (IM) membrane [15–17]. Its presence and role in mitochondrial biology have been extensively documented, as it plays a key role in the preservation of mitochondrial function, morphology, and plasticity [17–21]. YME1L forms homo-oligomeric complexes within the IM [17–21]. Studies have elucidated YME1L’s involvement in the regulation of mitochondrial protein degradation, including lipid transferring proteins, IM translocation proteins, and the dynamin-like GTPase optic atrophy 1 (OPA1) [17–19, 22]. Notably, its depletion led to a spectrum of cellular changes [20]. This includes impaired cell proliferation, alterations in cristae morphology, induction of oxidative stress, and a reduction in rotenone-sensitive respiration [20]. Furthermore, the depletion of YME1L results in the accumulation of non-assembled respiratory chain subunits, exemplified by Ndufb6, ND1, and Cox4 [20].

Recent studies have elucidated the oncogenic potential of YME1L in various cancer types, such as pancreatic ductal adenocarcinoma (PDAC), glioma and non-small cell lung cancer (NSCLC) [23–25]. Despite these significant insights into YME1L’s role in cancer progression, its expression and possible functions in NPC have yet to be investigated.

## Materials and methods

### Chemicals and reagents

Puromycin, N-acetyl cysteine (NAC), ATP, polybrene and other chemicals were provided by Sigma Chemicals (St. Louis, MO). Antibodies, mRNA primers and assay kits for in vitro cell experiments were provided by Dr. Cao unless otherwise mentioned [24, 25]. The medium, fetal bovine serum (FBS), antibiotics, and various other cell culture reagents were provided by Hyclone Co. (Suzhou, China).

### Cells

The detailed protocols of isolation and primary culture of human-derived NPC cells were reported previously [26] and primary cancer cells were utilized in our previous study [26]. NPC cells in the present study were from tumor tissues of four primary patients, these cells were separately named as pNPC-1, pNPC-2, pNPC-3 and pNPC-4. The primary human nasal epithelial cells (HNEpC), pHNEpC-1 and pHNEpC-2 (from two donors), were reported early [27]. The protocols of using human cells were approved by the Ethics Committee of Soochow University, in according to the principles of Declaration of Helsinki. The written-informed consent was obtained from each participant.

### Human tissues

All primary NPC patients who provided tissues to this study were administrated at authors’ institution (First affiliated Hospital of Soochow University) and each provided the written-informed consent. As reported previously [27], NPC tumor tissues (“T”) and matched adjacent normal tissues (“N”) were obtained freshly at the time of tumor resection surgery and were separately carefully. Part of the tissue was stored in liquid nitrogen immediately. For immunohistochemistry (IHC) or immunofluorescence assays, tissues were paraffin embedded. The protocols were approved by the Ethics Committee of Soochow University, in according to the principles of Declaration of Helsinki.

### Immunohistochemistry (IHC)

In brief, the paraffin-embedded tissue sections underwent baking, dewaxing, and hydration. Tissue sections then underwent antigen retrieval using citric acid buffer. Following hydrogen peroxide blocking, the primary antibody was applied and incubated for 12 h. Subsequently, biotin-labeled IgG antibody was applied for 2 h, followed by streptavidin-HRP incubation. The tissue sections were then stained with diaminobenzidine (DAB) for visualization. For IHC data quantification, the IHC score was calculated by the multiplication of the intensity score by the percentage score. Intensity was graded on a scale from 0 to 3, with 0 indicating no staining, 1 for weak staining, 2 for moderate staining, and 3 for strong staining. The proportion of positive cells ranged from 0-100%.

### Tissue fluorescence staining

The paraffin-embedded tissue sections were subjected to baking, dewaxing, and hydration. To minimize nonspecific binding, goat serum was used to block the tissue slices for 20 min at 37 °C. The tissue slices were then incubated with the primary antibody for 12 h when necessary. Subsequently, fluorescein-labeled IgG antibody (or fluorescence dyes) were added for 2 h, followed by thorough washing and examination using a confocal microscope (Zeiss).

### Gene and protein detection

Western blotting, quantitative reverse transcription-polymerase chain reaction (qRT-PCR) assays were reported in detail in elsewhere [28–30]. The isolation of mitochondria was via a high-speed centrifugation protocol by a Pierce kit (Pierce Biotechnology, Rockford, IL).

### YME1L shRNA

Silencing of YME1L was performed using a lentiviral-mediated shRNA method. Primary human NPC cells or nasal epithelial cells were cultured in polybrene-containing medium supplemented with 10% FBS until reaching 65% confluence. Subsequently, cells were infected with lentivirus carrying three different shRNA sequences targeting YME1L: kdYME1L and kdYME1L (-s2), provided by Dr. Cao [24], as well as kdYME1L (-s3), provided by Genechem. Viral infection was allowed to proceed for 48 h, after which cells were switched to fresh complete medium. Puromycin (2.5 μg/mL) was introduced to select stable cell populations, and this selection process continued for five passages. The expression level of YME1L was assessed in the resulting stable cells. For control experiments, cells were infected with lentivirus encoding a scramble control non-sense shRNA, and the selection process was identical. Adeno-associated viruses (AAV) expressing YME1L shRNA or scramble control shRNA, also provided by Dr. Cao [24], were employed in animal studies.

### YME1L knockout

To achieve YME1L knockout (KO), primary human NPC cells were cultured in polybrene-containing medium and infected with lentivirus encoding Cas9 [clustered regularly interspaced short palindromic repeats (CRISPR)-associated protein 9]. Following selection with puromycin, stable cells with Cas9 were generated. These Cas9-expressing cells were subsequently infected with lentivirus containing the CRISPR-YME1L-KO construct, which included small-guide RNA (sgRNA) targeting YME1L, including koYME1L provided by Dr. Cao [24] and koYME1L (sg2, provided by Genechem). Puromycin selection was applied for five passages to establish stable cell populations, which were then distributed into 96-well plates. *YME1L* KO was confirmed by PCR screening, and single stable cell clones were obtained. Control experiments involved stable transduction of cells with lentiviral Cas9-expressing construct along with lentiviral CRISPR-KO construct carrying non-sense sgRNA sequence.

### YME1L overexpression

For YME1L overexpression, primary human NPC cells were cultured in polybrene-containing medium and infected with lentivirus harboring the YME1L-expressing sequence [24] for 48 h. Following viral infection, cells were transferred to fresh complete medium, and puromycin (2.5 μg/mL) was added to the medium for selection of stable cells, which continued for 5-6 passages. The overexpression of YME1L, both at the mRNA and protein level, was consistently verified in the established stable cell populations (“oeYME1L”). Control experiments involved cells infected with lentivirus carrying an empty vector (“vec”), and the selection process was analogous.

### Cellular fluorescence dye assays

NPC cells were initially seeded to reach a confluence of 60–70%, and then specific treatments were employed. Following the designated incubation times, cells were subjected to fixation (with methanol), permeabilization (with Triton X-100), blocking (with BSA), and washing (with PBS). Subsequently, the cells were stained with fluorescence dyes, including CellROX, DCF-DA and BODIPY. Fluorescence images were captured using a Zeiss LSM980 microscope, and the intensity of the fluorescence was quantified using ImageJ software.

### The mitochondrial complex I activity and ATP assays

The enzymatic activity of mitochondrial complex I within mitochondrial fraction lysates was assessed using a commercial kit from Sigma. This kit utilizes spectrophotometry to track the conversion of NADH to NAD+ by complex I. The decrease in absorbance at 350 nm was recorded as a direct indicator of complex I activity. Cellular and tissue ATP contents were quantified using a commercial colorimetric kit, from Sigma, following the provided protocols. For each treatment, 20 μL of cellular or tissue lysates were analyzed.

### Oxygen consumption rate (OCR)

OCR was measured with an XF24 Extracellular Flux Analyzer (Agilent SeaHorse Bioscience) using the described protocols [31]. Cells were exposed sequentially to 1 μM oligomycin, 0.5 μM FCCP (carbonyl cyanide-p-trifluoromethoxyphenylhydrazone), and a mixture of 0.5 μM antimycin A plus Rotenone to assess basal OCR, ATP-linked OCR, maximal OCR, and non-mitochondrial OCR, respectively. The OCR measurements were normalized to the concentrations of intracellular proteins.

### Other in vitro assays

Cell proliferation (nuclear EdU staining), in vitro cell migration (“Transwell”) and cell invasion (“Matrigel Transwell”), cell viability (CCK-8 assay), Caspase-3 activity assay, Histone DNA ELISA, TUNEL-incorporated nuclei staining, Annexin V-FACS (fluorescence-activated cell sorting), propidium iodide (PI)-FACS assay of cell cycle progression, mitochondrial depolarization (JC-1 staining assays) and cell death (trypan blue staining) were described in detail in our previous study [27] or elsewhere [24, 25].

### Animal studies

In xenograft studies, four- to five-week-old nude mice, evenly distributed by gender and weighing 18.4–18.9 g as reported earlier [27], received subcutaneous (*s.c*.) injections of six million pNPC-1 cells suspended in 0.2 mL of Matrigel-containing serum-free medium per mouse. This led to the formation of pNPC-1 xenografts within 21 days, each reaching a volume of close to 100 mm^3^. These mice then underwent intratumoral injections of the specified adeno-associated viruses (AAV) (1.5 μL virus per xenograft, 1.0 × 10^9^ PFU, administered twice, 48 h apart). Tumor dimensions were measured and volumes estimated using the previously described formula [27]. Ethical approval for all animal procedures was granted by the Institutional Animal Care and Use Committee (IACUC) and the Ethics Board of Soochow University.

### Measuring the ratio of reduced glutathione (GSH) to oxidized glutathione (GSSG)

A GSH/GSSG ratio kit was obtained from Thermo Fisher Scientific (Suzhou, China). In brief, the xenograft tissue lysates were mixed with 5,5’-Dithio-bis(2-nitrobenzoic acid) (DTNB), glutathione reductase, and NADPH mix. Afterwards, the lysates were further mixed with the reaction solution, then the absorbance at 412 nm measured over five minutes under a spectrophotometer. A standard curve utilizing GSH and GSSG standards was created to determine their concentrations within the lysates and the ratio was normalized to the protein concentration for accurate comparison.

### Thiobarbituric acid reactive substances (TBAR) assay

Measuring the intensity of TBAR in tissue lysates is a common method for assessing lipid peroxidation. A TBAR assay kit was obtained from Thermo Fisher Scientific (Suzhou, China). TBAR intensity in tissue lysates was measured by first homogenizing tissue samples in a cold buffer, precipitating proteins with trichloroacetic acid (TCA), and allowing the supernatant to react with thiobarbituric acid (TBA) to form the TBAR complex. After cooling and centrifugation to remove any precipitate, the absorbance at 532 nm is measured using a spectrophotometer.

### Statistical analysis

The numerical data were with normal distribution and were reported as mean ± standard deviation (SD). To evaluate differences among three or more groups, one-way analysis of variance (ANOVA) followed by Tukey’s multiple comparison test, employing GraphPad Prism 5.01 software, was conducted. For the comparison of two groups, the Student’s *t* test was employed using Microsoft Excel 2013. Statistical significance was defined as a P-value less than 0.05.

## Results

### YME1L overexpression in NPC tissues and cells

**Fig. 1:**
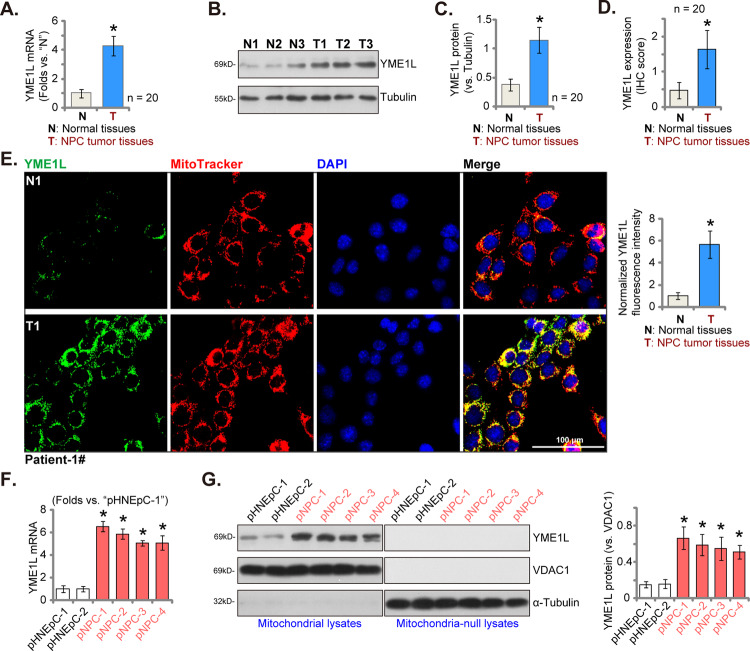
YME1L overexpression in NPC tissues and cells. *YME1L* mRNA (**A**) and protein (**B** and **C**) expression in NPC tumor tissues (“T”, *n* = 20) and their corresponding matched adjacent normal nasopharynx epithelial tissues (“N”, *n* = 20) were displayed, with quantified results provided. The immunohistochemistry (IHC) images depicting YME1L expression in the described human tissues were quantified, with IHC score results provided (**D**). Tissue immunofluorescence images, displaying YME1L (green fluorescence), the mitochondrial marker MitoTracker (red fluorescence), and nuclear DAPI staining (blue fluorescence) within the NPC tumor tissue and the adjacent normal epithelial tissue of Patient-1# were presented (**E**), and the normalized fluorescence intensity was quantified (**E**). *YME1L* mRNA expression (**F**) and protein expression (in both mitochondrial fraction lysates and mitochondria-null lysates **G**) were demonstrated in primary human NPC cells (“pNPC-1”, “pNPC-2”, “pNPC-3”, and “pNPC-4”) and primary human nasal epithelial cells (“pHNEpC-1” and “pHNEpC-2”) as described. The numerical values were mean ± standard deviation (SD). *P < 0.05 *vs*. “N” tissues or “pHNEpC-1” cells. Scale bar = 100 μm.

We first conducted an examination of YME1L expression within the NPC tissues of patients who had undergone administration of therapeutic interventions [27]. Both NPC tumor tissues (“T”) and the corresponding adjacent normal nasopharynx epithelial tissues (“N”) were from a cohort comprising twenty (*n* = 20) primary NPC patients [27]. Our findings revealed a notable elevation in *YME1L* mRNA expression levels within the tumor tissues, with a remarkable over four-fold increase compared to the adjacent normal tissues (Fig. 1A). Substantiating this observation, Western blotting analysis presented in Fig. 1B confirmed the upregulation of YME1L protein in NPC tumor tissues from three representative patients, denoted as “Patient-1#” to “Patient-3#”. Furthermore, quantified results derived from analysis of all twenty sets of Western blot data unequivocally substantiated the significant upregulation of YME1L protein in NPC tissue (*P* < 0.05 compared to “N” tissues, Fig. 1C). To further validate these findings, the quantified immunohistochemistry (IHC) images summarizing all twenty pairs of tissues’ results further corroborated significant YME1L protein upregulation in the NPC tissues (Fig. 1D).

To elucidate the subcellular localization of YME1L, we conducted tissue immunofluorescence assays utilizing a previously established protocol [25, 32]. As shown in Fig. 1E, YME1L protein, represented by green fluorescence, displayed clear co-localization with the mitochondrial marker MitoTracker, visualized in red fluorescence, within both the NPC tumor section (“T1”) and the corresponding adjacent normal lung epithelial tissue section (“N1”) from “Patient-1#”. The green fluorescence intensity of YME1L in NPC tumor sections markedly exceeded that observed in adjacent epithelial tissue (Fig. 1E). The intensity of YME1L green fluorescence was also quantified across 15 random microscope views of tissue sections from three representative patients (“Patient-1#“ to “Patient-3#“), and this intensity was normalized against the total count of cell nuclei. The findings further demonstrated a significant upregulation of mitochondrial YME1L in NPC tissues (Fig. 1E).

Additionally, we assessed YME1L expression across different NPC cell lines. Employing the described methodology [27], primary human NPC cells derived from four distinct patients (“Patient-1#” to “Patient-4#”), designated as “pNPC-1”, “pNPC-2”, “pNPC-3”, and “pNPC-4”, were isolated. mRNA expression of YME1L was substantially upregulated in all primary NPC cells (Fig. 1F). Conversely, in primary human nasal epithelial cells (HNEpC) obtained from two donors, “pHNEpC-1” and “pHNEpC-2” (as reported [27]), *YME1L* mRNA expression levels remained relatively low (Fig. 1F). Subsequently, mitochondrial fraction lysates from NPC cells and epithelial cells were segregated. YME1L protein was detected in the mitochondrial lysates alongside the voltage-dependent anion channel 1 (VDAC1), a mitochondrial marker (Fig. 1G). YME1L exhibited upregulation in primary NPC cells (Fig. 1G), while its expression remained low in epithelial cells (Fig. 1G). Notably, YME1L protein was absent in mitochondria-null lysates, as indicated by the presence of α-Tubulin (Fig. 1G) and the absence of VDAC1 (Fig. 1G).

### YME1L depletion led to damage of mitochondrial function in primary human NPC cells

**Fig. 2:**
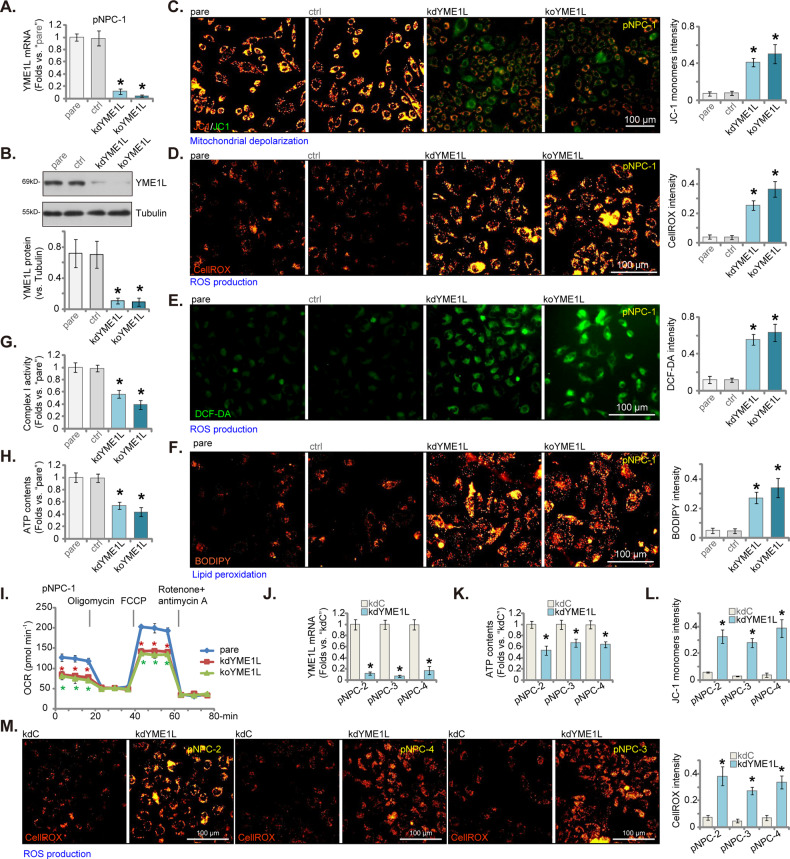
YME1L depletion led to damage of mitochondrial function in primary human NPC cells. The stable pNPC-1 primary NPC cells with the lentiviral YME1L shRNA (“kdYME1L”), the lentiviral CRISPR-YME1L-KO construct (koYME1L), or the lentiviral scramble control shRNA plus the lentiviral CRISPR-KO control treatment (“ctrl”) were established and expression YME1L (mRNA and protein) was examined (**A** and **B**). The exact same number of the above pNPC-1 cells were further cultivated for 36 h, mitochondrial depolarization (via testing JC-1 green monomer intensity, **C**), ROS levels (by measuring CellROX and DCF-DA fluorescence intensity, **D** and **E**) and lipid peroxidation (via testing BODIPY intensity, **F**) as well as the mitochondrial complex-1 activity (**G**) and ATP contents (**H**) were tested. The seahorse assay was performed to measure the oxygen consumption rate (OCR) (**I**). Primary NPC cells that were derived from other patients, pNPC-2/-3/-4, were stably transduced with the lentiviral YME1L shRNA (“kdYME1L”) or the lentiviral scramble control shRNA (“kdC”), relative expression of *YME1L* mRNA was shown (**J**); Cells were further cultivated for 36 h, cellular ATP content (**K**), mitochondrial depolarization (**L**), ROS levels (via measuring CellROX fluorescence intensity, **M**) were tested similarly. The numerical values were mean ± standard deviation (SD, *n* = 5). “pare” indicates the parental control cells. *P < 0.05 *vs*. “pare”/“kdC” cells. Experiments in this figure were repeated five times, and each time similar results obtained. Scale bar = 100 μm.

The current study aimed to understand the potential role of YME1L in NPC cells. Genetic approaches were utilized to downregulate YME1L expression in NPC cells. The lentivirus encoding YME1L shRNA (reported previously [23, 24]) was added to pNPC-1 primary cells and stable cells, “kdYME1L”, were selected following puromycin treatment. Alternatively, stable pNPC-1 cells-expressing Cas9 construct were further transduced with the lentiviral CRISPR-YME1L-KO construct, and single stable cells were selected by puromycin. These cells were named “koYME1L” cells. As control (“ctrl”), pNPC-1 cells were treated with lentiviral scramble control shRNA (“sh-C”) plus the lentiviral CRISPR-KO control (with non-sense sgRNA). As shown, *YME1L* mRNA (Fig. 2A) and protein (Fig. 2B) expression was substantially decreased in kdYME1L and koYME1L pNPC-1 cells.

YME1L knockdown or KO induced mitochondrial depolarization, causing JC-1 transition from red fluorescent aggregates to green fluorescent monomers (Fig. 2C). Moreover, YME1L depletion in pNPC-1 cells led to robust reactive oxygen species (ROS) production, which was evidenced by increased CellROX red fluorescence intensity (Fig. 2D) and DCF-DA green fluorescence intensity (Fig. 2E). In addition, increased BODIPY fluorescence demonstrated lipid peroxidation in YME1L-depleted pNPC-1 cells (Fig. 2F). Further supporting damage to mitochondrial function, we found that the mitochondrial complex-1 activity was decreased in kdYME1L pNPC-1 cells and koYME1L pNPC-1 cells (Fig. 2G), where ATP content was also reduced (Fig. 2H). The seahorse assay results further showed that shRNA-induced silencing or CRISPR/Cas9-induced KO of YME1L largely inhibited basal and maximal OCR in pNPC-1 cells (Fig. 2I). These results support that YME1L depletion causes considerable mitochondrial damage in primary pNPC-1 cells. The “ctrl” treatment, as expected, failed to significantly alter YME1L expression (Fig. 2A, B) or disrupt mitochondrial function (Fig. 2C–I) in pNPC-1 cells.

Two additional lentiviral shRNAs, kdYME1L (-s2) and kdYME1L (-s3), which target distinct sequences of YME1L, along with a separate lentiviral construct, koYME1L (sg2), that expresses sgRNA targeting another sequence of YME1L, also effectively depleted YME1L protein in pNPC-1 cells (Supplementary Fig. S2A). Consequently, this led to mitochondrial depolarization (evidenced by the accumulation of JC-1 green monomers, Supplementary Fig. S2B), ROS production (CellROX intensity increasing, Supplementary Fig. S2C), and enhanced lipid peroxidation (BODIPY fluorescence intensity rising, Supplementary Fig. S2D).

We next tested whether YME1L depletion led to similar effects in other NPC cells. Primary human NPC cells, derived from three other patients, pNPC-2/-3/-4, were infected with lentivirus encoding YME1L shRNA. Stable cells (“kdYME1L”) were established by puromycin selection, resulting in downregulated *YME1L* mRNA expression (Fig. 2J). In the primary NPC cells, YME1L knockdown similarly caused ATP reduction (Fig. 2K), mitochondrial depolarization (accumulation of JC-1 green monomers, Fig. 2L) and ROS production (CellROX intensity increasing, Fig. 2M).

### Apoptosis activation after YME1L depletion in primary human NPC cells

**Fig. 3:**
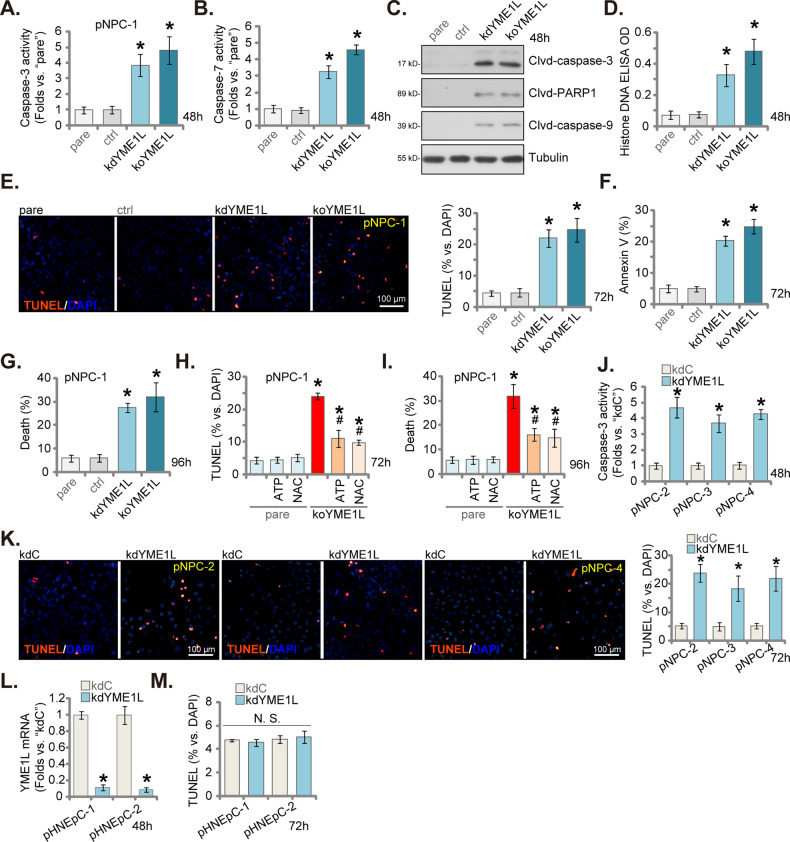
Apoptosis activation after YME1L depletion in primary human NPC cells. The stable pNPC-1 primary NPC cells with the lentiviral YME1L shRNA (“kdYME1L”), the lentiviral CRISPR-YME1L-KO construct (koYME1L), or the lentiviral scramble control shRNA plus the lentiviral CRISPR-KO control treatment (“ctrl”) were established, the exact same number of the above pNPC-1 cells were cultivated for designated hours, Caspase-3 and Caspase-7 activities (**A** and **B**), expression of apoptosis-associated proteins (**C**) and Histone DNA contents (**D**) were measured. Cell apoptosis was tested via nuclear TUNEL incorporation fluorescence staining assay (**E**) and Annexin V-PI FACS (**F**). Cell death was measured by trypan blue staining assays, with results quantified (**G**). The ctrl pNPC-1 cells or the koYME1L pNPC-1 cells were treated with the antioxidant NAC (0.5 mM) or ATP (1 mM) for designated hours, cell apoptosis (TUNEL assays, **H**) and death (trypan blue assays, **I**) were measured. Primary NPC cells that were derived from other patients, pNPC-2/-3/-4, or the primary human nasal epithelial cells (pHNEpC-1 and pHNEpC-2, derived from two donors) were stably transduced with the lentiviral YME1L shRNA (“kdYME1L”) or the lentiviral scramble control shRNA (“kdC”); The exact same number of cells were further cultivated for designated hours, the Caspase-3 activity (**J**) and apoptosis (TUNEL assays, **K** and **M**) were measured similarly. The relative *YME1L* mRNA expression in the epithelial cells was shown (**L**). The numerical values were mean ± standard deviation (SD, *n* = 5). “pare” indicates the parental control cells. *P < 0.05 *vs*. “pare”/ “kdC” cells. ^#^ *P* < 0.05 *vs*. “koYME1L” cells (**H** and **I**). “N. S.” stands for non*-*statistical difference (*P* > 0.05). Experiments in this figure were repeated five times, and each time similar results obtained. Scale bar = 100 μm.

Following mitochondrial damage, the caspase-dependent apoptosis cascade can be induced [20, 23, 24]. Here, the caspase-3 (Fig. 3A) and caspase-7 activity (Fig. 3B) were both significantly augmented in kdYME1L pNPC-1 cells and koYME1L pNPC-1 cells. Moreover, cleavage of caspase-3, caspase-9 and poly(ADP-ribose) polymerase 1 (PARP1) were induced following YME1L depletion in pNPC-1 cells (Fig. 3C). The content of histone-bound DNA, the characteristic marker of apoptosis activation [33, 34], was increased in pNPC-1 cells with YME1L knockdown or KO (Fig. 3D). Validating apoptosis activation, we found that the percentage of TUNEL-stained nuclei was significantly increased in kdYME1L pNPC-1 cells and koYME1L pNPC-1 cells (Fig. 3E). Moreover, Annexin V-sorted pNPC-1 cell numbers were substantially increased with YME1L depletion (Fig. 3F). Moreover, we found that YME1L silencing or KO induced pNPC-1 cell death and increased trypan blue staining (Fig. 3G). Importantly, we provided evidence to support that damage to mitochondrial function could be an important mechanism of YME1L depletion-induced cytotoxicity in NPC cells. The powerful antioxidant N-acetyl cysteine (NAC) and ATP supplement ameliorated koYME1L-induced apoptosis (Fig. 3H) and death (Fig. 3I) of pNPC-1 cells.

Additionally, the suppression of YME1L protein through kdYME1L (-s2) and kdYME1L (-s3), as well as KO via koYME1L (sg2), triggered apoptosis activation in pNPC-1 cells, as indicated by an elevated TUNEL-nuclei ratio (Supplementary Fig. S2E). In other patients-derived primary NPC cells, pNPC-2/-3/-4, YME1L silencing by kdYME1L (see Fig. 2) similarly augmented caspase-3 activity (Fig. 3J) and increased TUNEL-incorporated nuclei percentage (Fig. 3K), indicating activation of the caspase-apoptosis cascade. In pHNEpC-1 and pHNEpC-2 nasal epithelial cells (as reported previously [27]), infection of lentivirus encoding YME1L shRNA (“kdYME1L”) similarly downregulated *YME1L* mRNA expression (Fig. 3L), but failed to provoke apoptosis, as the TUNEL-stained nuclei percentage remained unaltered (Fig. 3M).

### YME1L depletion inhibits proliferation, viability, cell cycle progression and migration of primary human NPC cells

**Fig. 4:**
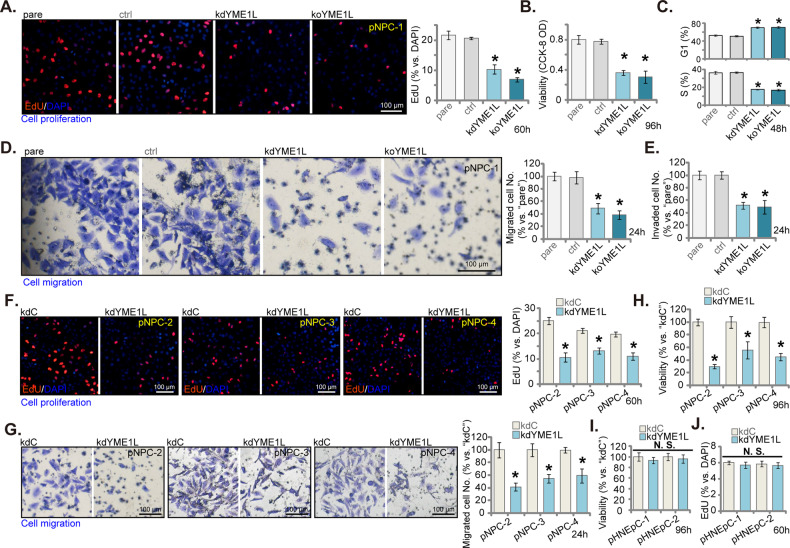
YME1L depletion inhibits proliferation, viability, cell cycle progression and migration of primary human NPC cells. The stable pNPC-1 primary NPC cells with the lentiviral YME1L shRNA (“kdYME1L”), the lentiviral CRISPR-YME1L-KO construct (koYME1L), or the lentiviral scramble control shRNA plus the lentiviral CRISPR-KO control treatment (“ctrl”) were established, the exact same number of the above pNPC-1 cells were cultivated for designated hours, cell proliferation, viability, cell cycle progression, migration, invasion were tested via nuclear EdU fluorescence staining (**A**), CCK-8 (**B**), PI-FACS (**C**), “Transwell” (**D**) and “Matrigel Transwell” (**E**) assays, respectively. Primary NPC cells that were derived from other patients, pNPC-2/-3/-4, or the primary human nasal epithelial cells (pHNEpC-1 and pHNEpC-2, derived from two donors) were stably transduced with the lentiviral YME1L shRNA (“kdYME1L”) or the lentiviral scramble control shRNA (“kdC”); The exact same number of cells were further cultivated for designated hours, cell proliferation (**F** and **J**), migration (**G**) and viability (**H** and **I**) were tested similarly. The numerical values were mean ± standard deviation (SD, *n* = 5). “pare” indicates the parental control cells. *P < 0.05 *vs*. “pare”/“kdC” cells. “N. S.” stands for non-statistical difference (*P* > 0.05). Experiments in this figure were repeated five times, and each time similar results obtained. Scale bar = 100 μm.

We next explored the potential role of YME1L depletion on the function of NPC cells. As shown, in kdYME1L pNPC-1 cells and koYME1L pNPC-1 cells, the percentage of EdU-incorporated nuclei was largely decreased (Fig. 4A), showing that YME1L depletion inhibited pNPC-1 cell proliferation. YME1L silencing or KO also decreased the viability of pNPC-1 cells, which was demonstrated by reduced CCK-8 optical density (OD, Fig. 4B). In addition, YME1L depletion caused G1-S cell cycle arrest, as the G1-phase percentage was increased whereas the S-phase percentage was decreased in kdYME1L pNPC-1 cells and koYME1L pNPC-1 cells (Fig. 4C). Moreover, based on the results from “Transwell” assays, pNPC-1 in vitro cell migration (Fig. 4D) and invasion (Fig. 4E) were largely inhibited by YME1L depletion. The “ctrl” (see Figs. 2 and 3) failed to significantly alter pNPC-1 cellular function (Fig. 4A–E). Furthermore, the depletion of YME1L through kdYME1L (-s2) and kdYME1L (-s3), or koYME1L (sg2), significantly reduced pNPC-1 cell proliferation (measured by nuclear EdU incorporation, Supplementary Fig. S2F), migration (Supplementary Fig. S2G), and invasion (Supplementary Fig. S2H).

In other primary NPC cells, pNPC-2/-3/-4, YME1L silencing by kdYME1L (see Figs. 2 and 3) similarly inhibited cell proliferation and decreased the percentage of EdU nuclei (Fig. 4F). Cell migration, tested via “Transwell” assay, was largely suppressed in kdYME1L primary NPC cells (Fig. 4G). Silencing of YME1L also decreased viability (CCK-8 OD) in pNPC-2/-3/-4 cells (Fig. 4H). In contrast, YME1L silencing in pHNEpC-1 and pHNEpC-2 epithelial cells (see Fig. 3) failed to inhibit cell viability and proliferation, which were respectively measured by CCK-8 (Fig. 4I) and nuclear EdU incorporation (Fig. 4J) assays.

### YME1L overexpression induces pro-tumorigenic activity in primary human NPC cells

**Fig. 5:**
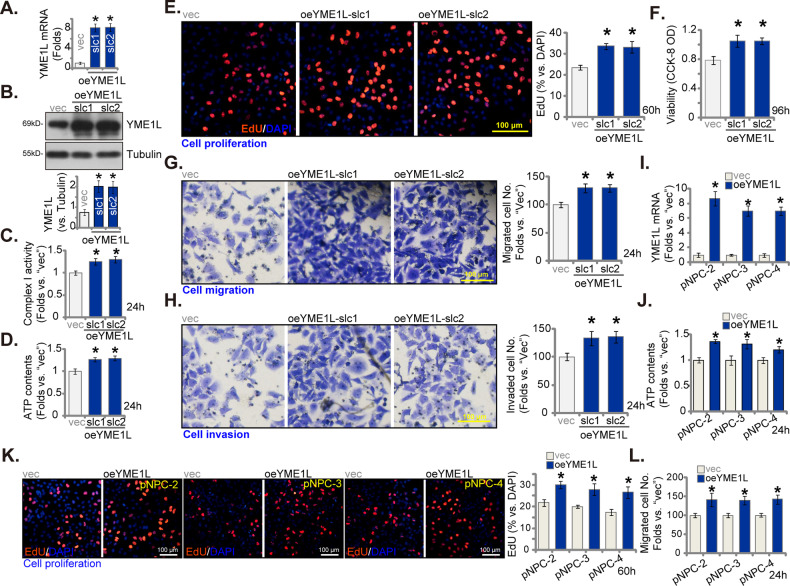
YME1L overexpression induces pro-tumorigenic activity in primary human NPC cells. The stable pNPC-1 primary NPC cells with the lentiviral YME1L-expressing construct (oeYME1L-slc1 and oeYME1L-slc2, two stable selections) or the vector (“vec”) were established and expression YME1L (mRNA and protein) was examined (**A** and **B**). The exact same number of the above pNPC-1 cells were further cultivated for designated hours, the mitochondrial complex-1 activity (**C**) and ATP contents (**D**) were tested; Cell proliferation, viability, migration and invasion were tested via nuclear EdU fluorescence staining (**E**), CCK-8 (**F**), “Transwell” (**G**), and “Matrigel Transwell” (**H**) assays, respectively. Primary NPC cells that were derived from other patients, pNPC-2/-3/-4, with the lentiviral YME1L-expressing construct (“oeYME1L”) or the vector (“vec”) were formed and expression of *YME1L* mRNA was tested (**I**); ATP contents (**J**), cell proliferation (**K**) and migration (**L**) were tested similarly. The numerical values were mean ± standard deviation (SD, *n* = 5). *P < 0.05 *vs*. “vec” cells. Experiments in this figure were repeated five times, and each time similar results obtained. Scale bar = 100 μm.

The aforementioned findings demonstrate that reducing the expression of YME1L, either through knockdown or knockout, impaired mitochondrial function and exhibited anti-cancer properties in NPC cells. In light of these observations, we postulated that forced YME1L overexpression might promote tumorigenic behaviors. To investigate this hypothesis, we introduced a lentivirus encoding the YME1L-expressing construct into pNPC-1 primary cells. Subsequently, puromycin was employed to establish two stable pNPC-1 cell selections, designated as oeYME1L-slc1 and oeYME1L-slc2. The levels of *YME1L* mRNA (Fig. 5A) and protein (Fig. 5B) were significantly elevated in oeYME1L pNPC-1 cells. Concomitantly, the activity of mitochondrial complex-1 (Fig. 5C) and ATP content (Fig. 5D) were substantially increased. Thus, the ectopic overexpression of YME1L heightened mitochondrial activity in primary NPC cells.

Further functional investigation revealed that YME1L overexpression promoted nuclear EdU incorporation and augmented the proliferation of pNPC-1 cells (Fig. 5E). Additionally, the CCK-8 viability OD of pNPC-1 cells exhibited an increase (Fig. 5F). Furthermore, in vitro cell migration and invasion of pNPC-1 cells were notably accelerated following YME1L overexpression (Fig. 5G, H). In the case of other primary NPC cells, pNPC-2/-3/-4, stable transduction of the lentiviral YME1L-expressing construct (“oeYME1L”) also led to an increase in YME1L expression (Fig. 5I). In keeping with the outcomes in pNPC-1 cells, oeYME1L heightened ATP levels (Fig. 5J), enhanced cell proliferation (Fig. 5K) and migration (Fig. 5L) in the primary NPC cells. Collectively, these results substantiate that YME1L overexpression induces a pro-tumorigenic phenotypes in primary human NPC cells.

### YME1L is important for Akt-mTOR in NPC cells

**Fig. 6:**
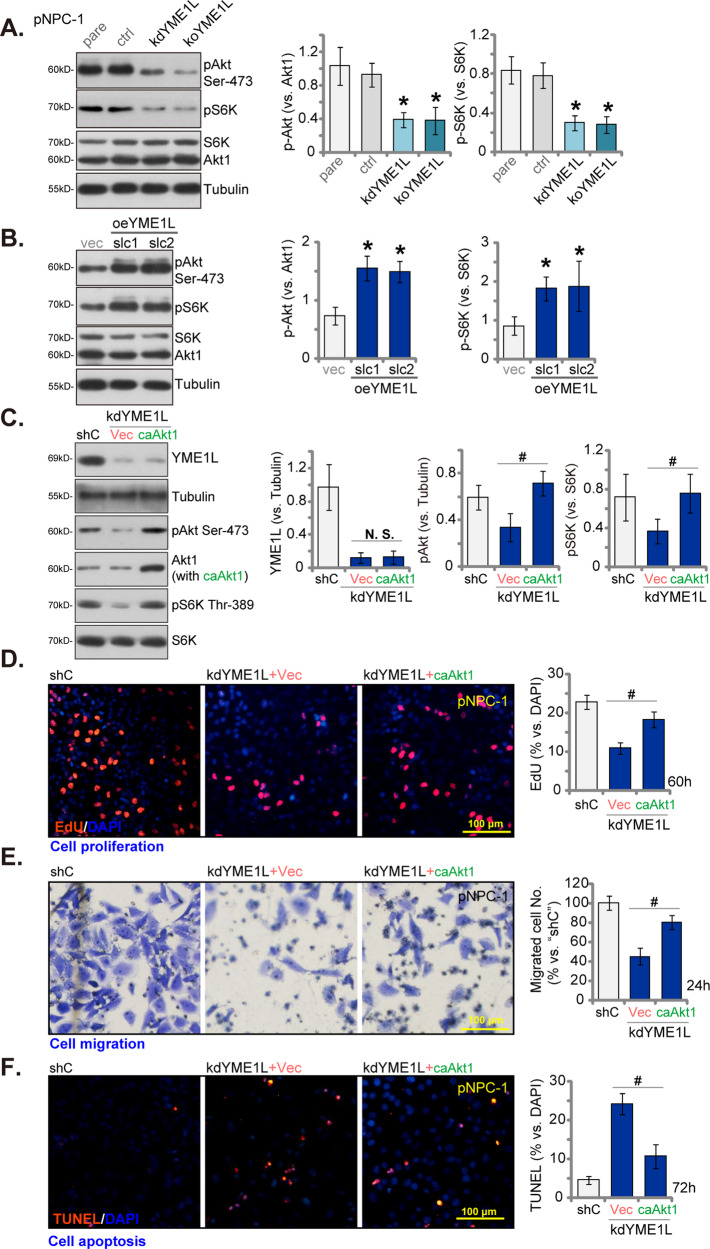
YME1L is important for Akt-mTOR activation in NPC cells. The stable pNPC-1 primary NPC cells with the lentiviral YME1L shRNA (“kdYME1L”), the lentiviral CRISPR-YME1L-KO construct (koYME1L), or the lentiviral scramble control shRNA plus the lentiviral CRISPR-KO control treatment (“ctrl”), the lentiviral YME1L-expressing construct (oeYME1L-slc1 and oeYME1L-slc2, two stable selections) or the vector (“vec”) were cultivated for 12 h, expression of listed proteins was tested (**A** and **B**). kdYME1L pNPC-1 cells were further stably transduced with a S473D constitutively-active mutant Akt1 (caAkt1) or the empty vector (“Vec”), expression of listed proteins was shown (**C**). These cells were further cultivated for designated hours, cell proliferation, migration and apoptosis were examined via EdU-nuclei staining (**D**), “Transwell” (**E**) and TUNEL-nuclei (**F**) assays, respectively. The numerical values were mean ± standard deviation (SD, *n* = 5). * *P* < 0.05 *vs*. “pare”/“vec” cells (A and **B**). ^#^ *P* < 0.05 (**C**–**F**). “N. S.” stands for non-statistical difference (*P* > 0.05).Experiments in this figure were repeated five times, and each time similar results obtained. Scale bar = 100 μm.

Given the established role of Akt-mTOR overactivation in the initiation and advancement of NPC [4, 35, 36], our investigation aimed to elucidate the potential impact of YME1L on Akt-mTOR activation within NPC cells. In pNPC-1 cells, the depletion of YME1L, achieved through lentiviral YME1L shRNA (kdYME1L) or the lentiviral CRISPR-YME1L-KO construct (koYME1L), resulted in a notable reduction in the phosphorylation of Akt (at Ser-473) and S6K (at Thr-389) (Fig. 6A). Importantly, the overall protein levels of Akt1 and S6K remained unchanged in these cells (Fig. 6A). Conversely, in pNPC-1 cells overexpressing YME1L (oeYME1L-slc1 and oeYME1L-slc1, as demonstrated in Fig. 5), there was an augmentation in Akt-S6K phosphorylation (Fig. 6B). These findings strongly support the pivotal role of YME1L in facilitating Akt-mTOR activation in NPC cells. To understand the connection between YME1L-driven NPC cell progression and the activation of the Akt-mTOR cascade, we introduced a S473D constitutively-active mutant of Akt1 (caAkt1) into YME1L-silenced pNPC-1 cells (kdYME1L). As demonstrated, caAkt1 effectively restored Akt-S6K phosphorylation levels without altering YME1L protein expression in kdYME1L pNPC-1 cells (Fig. 6C). Consequently, in pNPC-1 cells, caAkt1 significantly counteracted the inhibition of proliferation induced by YME1L silencing (tested via nuclear EdU assays, Fig. 6D), mitigated the reduction in cell migration (Fig. 6E), and attenuated apoptosis (Fig. 6F) caused by YME1L downregulation. These results provide robust evidence supporting that YME1L-driven NPC cell progression involves activation of the Akt-mTOR cascade.

### YME1L silencing impedes NPC xenograft growth in nude mice

**Fig. 7:**
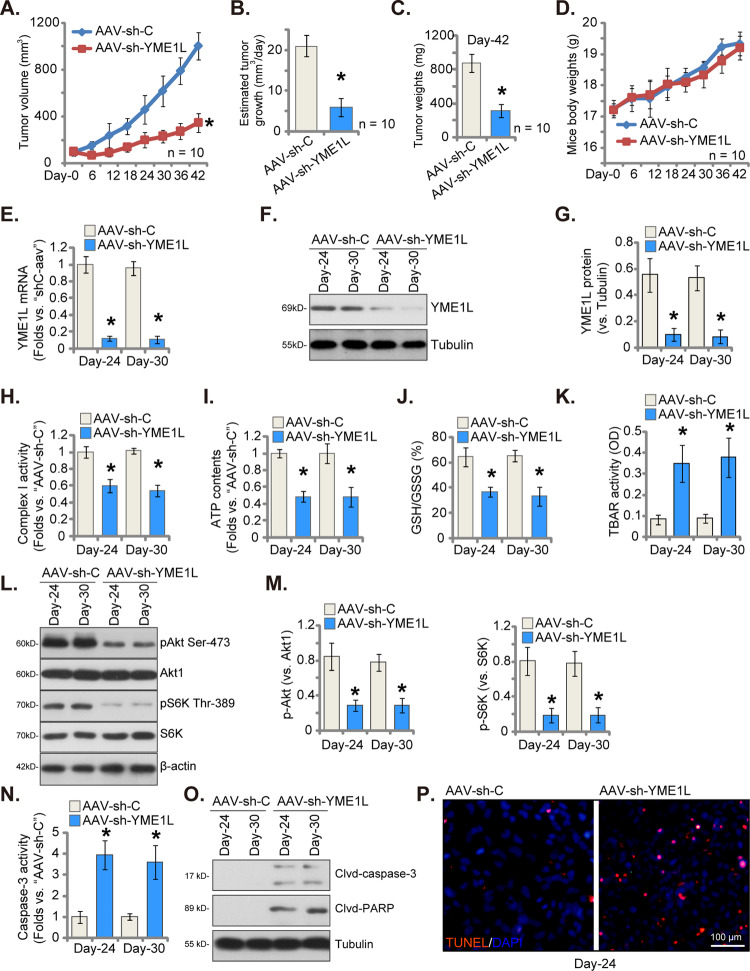
YME1L silencing impedes NPC xenograft growth in nude mice. The pNPC-1 xenograft-bearing nude mice were subject to intratumoral injection of the described virus, tumor volumes (in mm^3^, **A**) and the animal weights (in **G**, **D**) were recorded every six days. The estimated daily pNPC-1 xenograft growth, in mm^3^ per day, was also shown (**B**). All pNPC-1 xenografts were isolated at Day-42 and weighted (**C**). Expression of *YME1L* mRNA and listed proteins in the described pNPC-1 xenograft tissues was tested (**E**–**G**, **L**, **M** and **O**). The mitochondrial complex-1 activity (**H**), ATP contents (**I**), GSH/GSSG ratio (**J**), TBAR activity (**K**) and Caspase-3 activity (**N**) in tissue lysates were examined as well. The pNPC-1 xenograft sections were also subject to fluorescence detection of TUNEL-positive nuclei (**P**). The data were presented as mean ± standard deviation (SD). In **A**–**D**, ten mice were in each group (*n* = 10). For **E**–**P**, five random tissue pieces in each xenograft were tested (*n* = 5). *P < 0.05 *vs*. “AAV-shC” group. Scale bar = 100 μm.

The potential role of YME1L on NPC cell growth in vivo was studied. Using the previously-described protocol [27], pNPC-1 primary cells, at 6 × 10 ^6^ cells (in serum free medium) per mouse, were *s.c*. injected to the flanks of the nude mice. Subcutaneous pNPC-1 xenografts formed 21 days after injection of cells (“Day-0”). Thereafter, pNPC-1 xenograft-bearing nude mice were assigned randomly into two groups of 10 mice per group (*n* = 10). One group received YME1L shRNA-expressing adeno-associated virus AAV (“AAV-sh-YME1L”) and the other group received scramble control shRNA-expressing AAV (“AAV-sh-C”). The virus was injected directly into the pNPC-1 xenografts and was injected twice, with a 48 h interval between injections. Tumor volumes were then recorded (every six days) using the described protocol [27]. As demonstrated, AAV-sh-YME1L injection significantly impeded pNPC-1 xenograft growth in nude mice and the volumes of AAV-sh-YME1L-treated pNPC-1 xenografts were substantially lower than those with AAV-sh-C treatment (Fig. 7A). The estimated daily pNPC-1 xenograft growth, in mm^3^ per day, was remarkably decreased following AAV-sh-YME1L injection (Fig. 7B). Forty-two days after the first virus injection (“Day-42”), all pNPC-1 xenografts of the two groups were isolated and individually weighted. The AAV-sh-YME1L group pNPC-1 xenografts were significantly lighter than those with AAV-sh-C injection (Fig. 7C). There was, however, no significant difference in weights between these two groups of nude mice (Fig. 7D).

On the 24th (Day-24) and 30th (Day-30) days following the initial virus injection, we isolated a single pNPC-1 xenograft from each group, resulting in a total of four pNPC-1 xenografts. Subsequently, a portion of each xenograft was cut into pieces and homogenized. Our analysis revealed a significant reduction in both mRNA (Fig. 7E) and protein (Fig. 7F, G) expression of YME1L in the pNPC-1 xenograft tissues injected with the YME1L-shRNA virus. Furthermore, we observed notable alterations in mitochondrial function within the AAV-sh-YME1L-injected pNPC-1 xenograft tissues. Specifically, mitochondrial complex-1 activity (Fig. 7H), ATP levels (Fig. 7I), and the GSH/GSSG ratio (Fig. 7J) were all significantly decreased. This dysfunction was further supported by increased TBAR activity, indicative of lipid peroxidation, in the YME1L-silenced xenograft tissues (Fig. 7K). Moreover, the phosphorylation of Akt-S6K1 was reduced in pNPC-1 xenograft tissues with YME1L silencing (Fig. 7L, M), indicating inhibition of the Akt-mTOR pathway.

Further examination of the xenograft tissue revealed an enhancement in caspase-3 activity in the pNPC-1 xenograft tissues injected with AAV-sh-YME1L (Fig. 7N). This increase was associated with elevated levels of cleaved-caspase-3 and cleaved-PARP (Fig. 7O), indicative of apoptosis activation. Additionally, tissue fluorescence results provided further support for apoptosis activation in YME1L-silenced pNPC-1 xenografts, as evidenced by the augmented percentage of TUNEL-positive apoptotic nuclei (Fig. 7P). Collectively, these signaling outcomes demonstrate that AAV-sh-YME1L injection leads to YME1L silencing, mitochondrial dysfunction, Akt-mTOR pathway inhibition, and apoptosis induction in pNPC-1 xenografts.

## Discussion

Liu et al., demonstrated that YME1L expression is significantly elevated in glioma tissues and different glioma cells [24]. YME1L depletion, using the shRNA strategy or the CRISPR/Cas9 gene editing method, disrupted mitochondrial function in glioma cells, causing mitochondrial depolarization, reactive oxygen species production and lipid peroxidation, leading to reduced cell viability, proliferation and migration of glioma cells [24]. Conversely, forced overexpression of YME1L enhanced mitochondrial function and augmented glioma cell proliferation and migration [24]. In vivo, the subcutaneous and orthotopic growth of primary glioma xenografts in nude mice was largely inhibited by YME1L shRNA or knockout [24]. In addition, the same group reported that YME1L expression driven by the mitochondrial protein TIMM44 (translocase of inner mitochondrial membrane 44) promoted glioma cell growth [25]. MacVicar et al., reported that YME1L-mediated remodeling of the mitochondrial proteome was important for the growth of pancreatic ductal adenocarcinoma (PDAC) cells in both spheroid and xenograft models [16]. Xia et al., reported that YME1L is overexpressed in non-small cell lung cancer (NSCLC) tissue and cells, where it promotes tumor growth while inhibiting apoptosis. Silencing or knocking out YME1L in NSCLC cells led to suppressed cell proliferation and enhanced apoptosis through mitochondrial dysfunction, whereas overexpression enhanced tumorigenic activities [23].

**Fig. 8:**
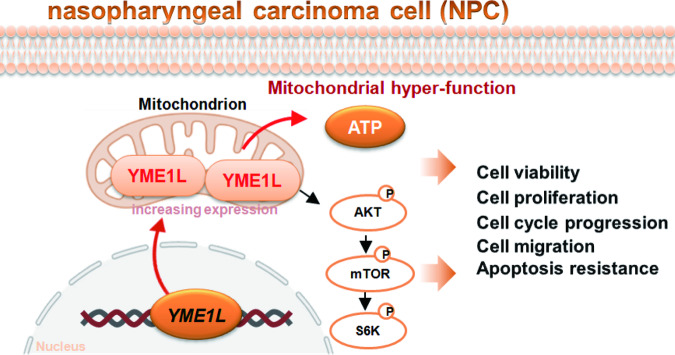
Overexpressed YME1L promotes NPC cell growth possibly by maintaining mitochondrial hyper-function and promoting Akt-mTOR activation. The proposed signaling cartoon of the study.

The results of this study underscore the potential of YME1L as a promising therapeutic NPC target. We report a substantial upregulation of YME1L in NPC tissues obtained from patients and primary human NPC cells, whereas YME1L expression is relatively low in adjacent normal tissues and primary nasal epithelial cells. Depletion of YME1L, either through shRNA or KO, exerted pronounced inhibitory effects on primary NPC cells, impeding cell viability, proliferation, cell cycle progression, and migratory capabilities, concomitant with an elevation in caspase-mediated apoptosis. Conversely, ectopic YME1L expression conferred pro-tumorigenic properties, enhancing NPC cell proliferation and migration. In vivo studies demonstrated that intratumoral administration of AAV expressing YME1L-shRNA effectively curtailed the growth of subcutaneous NPC xenografts in nude mice. These collective observations suggest that YME1L holds promise as a therapeutic target warranting further exploration in NPC (Fig. 8).

The aberrant activation of the Akt-mTOR cascade constitutes a significant signaling aberration frequently associated with the pathogenesis and progression of NPC [4, 35]. Akt-mTOR hyperactivation not only facilitates uncontrolled cell proliferation but also confers a pro-survival milieu by antagonizing apoptosis, thereby promoting cell growth even under adverse conditions [4, 35, 37, 38]. Moreover, the heightened migratory and invasive capabilities of NPC cells are attributed to the activation of Akt-mTOR, enabling metastasis [4, 35, 37, 38]. As such, the Akt-mTOR signaling axis emerges as a critical player in the pathogenic landscape of NPC [4, 35, 37, 38].

EBV plays a pivotal role by directly or indirectly modulating cellular signaling pathways through EBV-encoded proteins [38]. Overexpression and activation of receptor tyrosine kinases (RTKs), genetic alterations in the phosphatidylinositol 3-kinase (PI3K) pathway components, a hypoxic tumor microenvironment, and chronic inflammation collectively contribute to Akt-mTOR pathway hyperactivation. Additionally, genetic alterations in upstream regulators including Akt amplification or *PTEN* depletion further promote constitutive pathway activation [4, 35, 37]. It is therefore extremely important to further explore the underlying mechanism of Akt-mTOR activation in NPC.

Our research sheds light on the crucial significance of YME1L in promoting Akt-mTOR activation in NPC cells. When YME1L levels were reduced by shRNA/KO, there was a noticeable decrease in the phosphorylation of Akt-S6K in primary NPC cells. Conversely, elevating YME1L expression led to an enhancement of Akt-S6K phosphorylation in primary NPC cells. The introduction of caAkt1 effectively restored Akt-S6K phosphorylation in YME1L-silenced NPC cells and counteracted the inhibitory effects of YME1L shRNA on cell proliferation, migration, and apoptosis. In vivo, Akt-mTOR inactivation was detected in YME1L-silenced NPC xenograft tissues. Liu et al., also reported that YME1L plays a critical role in regulating the expression of G alpha inhibitory subunit 1 (Gαi1) and downstream Akt-mTOR activation in glioma cells. Reductions in Gαi1 expression and subsequent Akt activation were observed following YME1L silencing or knockout. Conversely, YME1L overexpression in primary glioma cells led to enhanced levels of Gαi1 and increased Akt activation [24]. These results support that YME1L-driven NPC cell growth is mediated, at least in part, by promoting Akt-mTOR activation (Fig. 8).

The study has certain limitations. Specifically, the molecular mechanism through which YME1L promotes Akt-mTOR activation in NPC cells remains to be elucidated. Additionally, further research is required to understand the impact of YME1L overexpression on the mitochondrial proteome, morphology, and advanced functions in NPC cells. Also, the specific mechanisms by which YME1L is overexpressed in NPC have not been studied.

## Conclusion

In summary, this study demonstrated that YME1L, a key regulator of mitochondrial function, is significantly upregulated in NPC and plays a crucial role in NPC progression by influencing mitochondrial function, viability, proliferation, migration, and Akt-mTOR activation, with potential implications for NPC tumorigenesis.

### Supplementary information

Figure S1

## Data Availability

All data are available upon reasonable request.
