# Supplementary material for: Expression and functional implications of YME1L in nasopharyngeal carcinoma
Source: Cell Death Dis. 2024 Jun 18;15(6):423. doi: 10.1038/s41419-024-06811-6 (PMC11189534; doi:10.1038/s41419-024-06811-6)
Supplement: Supplementary file 1 — Figure S1 [file 41419_2024_6811_MOESM1_ESM.pdf]

Figure S1. The uncropped blotting images.

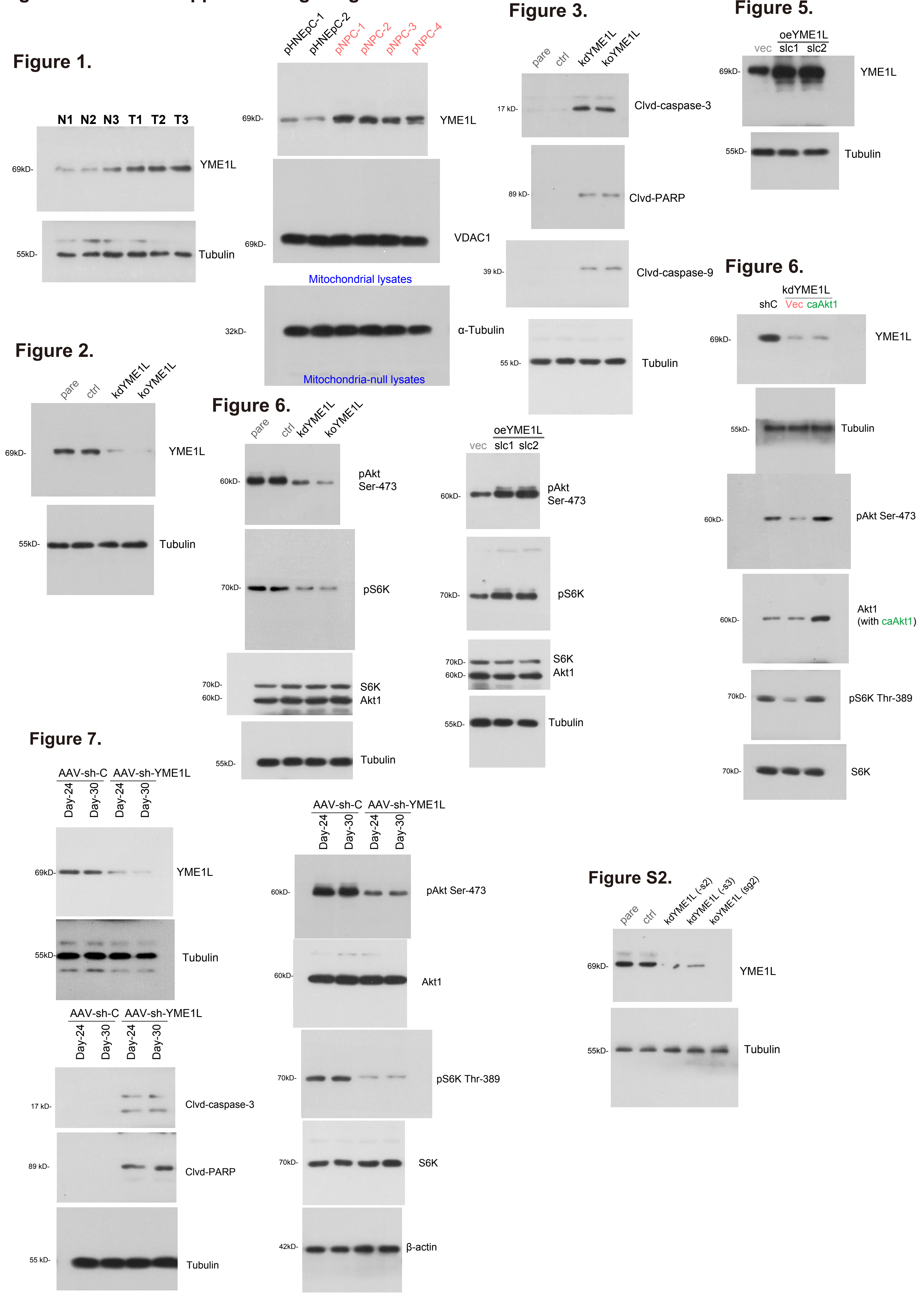

Figure S2.

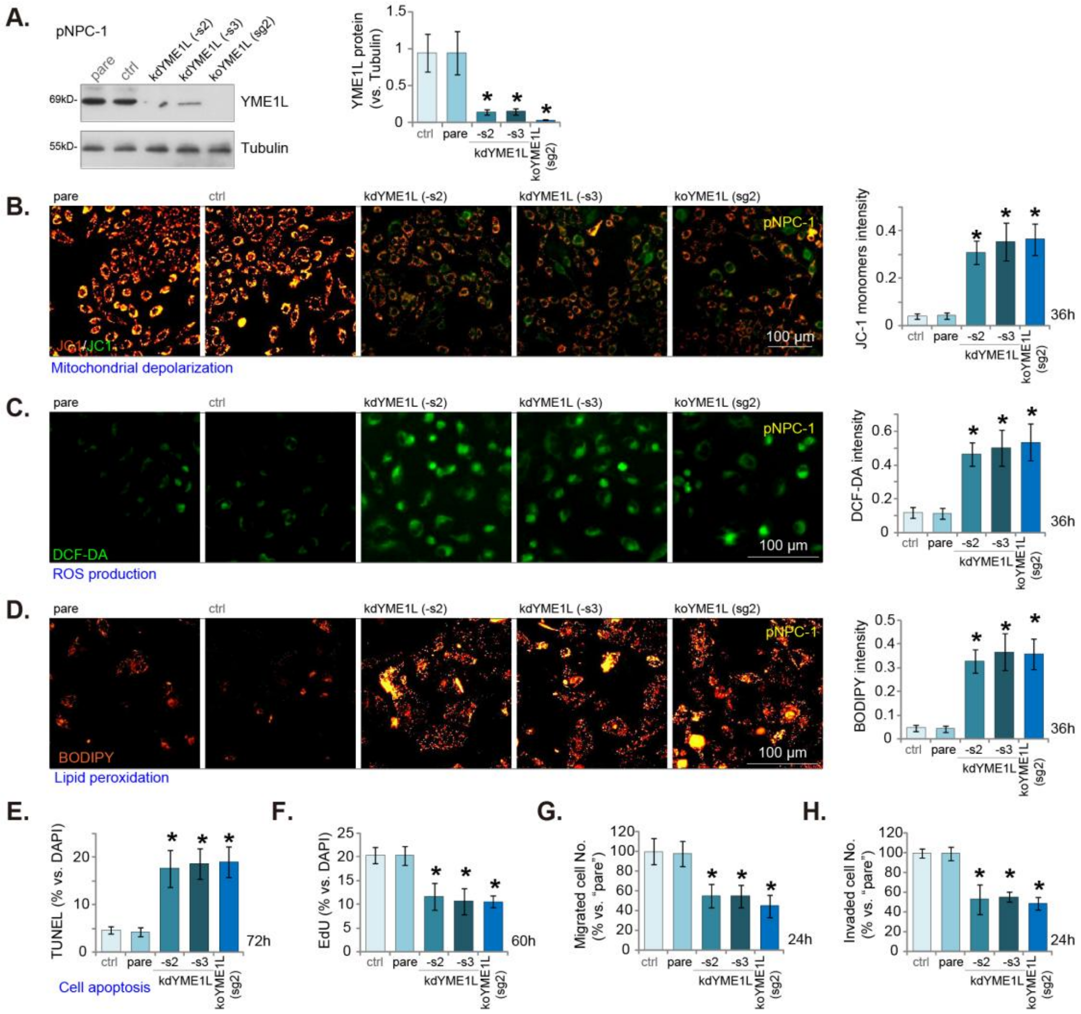

Figure S2. The stable pNPC-1 primary cells with the lentiviral YME1L shRNA [kdYME1L (-s2) or kdYME1L (-s3)], the lentiviral CRISPR-YME1L-KO construct [koYME1L (sg2)], or the lentiviral scramble control shRNA plus the lentiviral CRISPR-KO control treatment ("ctrl"), were established, and YME1L protein expression was examined (**A**). The same number of pNPC-1 cells were subsequently cultured for designated time periods to evaluate mitochondrial depolarization (by measuring JC-1 green monomer intensity, **B**), ROS levels (via DCF-DA fluorescence intensity, **C**), lipid peroxidation (through BODIPY intensity, **D**), and cell apoptosis (by analyzing TUNEL-nuclei ratio, **E**). Additionally, tests were conducted for cell proliferation (through measuring nuclear EdU incorporation, **F**), migration (**G**), and invasion (**H**).The numerical values were mean ± standard deviation (SD, n=5). "pare" indicates the parental control cells. * $\boldsymbol{P} < 0.05$ *vs.* "pare" cells. Experiments in this figure were repeated five times, and each time similar results obtained. Scale bar = 100 μm.
